# Chikungunya Outbreaks Caused by African Genotype, India

**DOI:** 10.3201/eid1210.060529

**Published:** 2006-10

**Authors:** Prasanna N. Yergolkar, Babasaheb V. Tandale, Vidya A. Arankalle, Padmakar S. Sathe, Sudeep A.B., Swati S. Gandhe, Mangesh D. Gokhle, George P. Jacob, Supriya L. Hundekar, Akhilesh C. Mishra

**Affiliations:** * National Institute of Virology, Pune, India

**Keywords:** Chikungunya epidemics, India, RT-PCR, genotypes, phylogenic analysis, dispatch

## Abstract

Chikungunya fever is reported in India after 32 years. Immunoglobulin M antibodies and virus isolation confirmed the cause. Phylogenic analysis based on partial sequences of NS4 and E1 genes showed that all earlier isolates (1963–1973) were Asian genotype, whereas the current and Yawat (2000) isolates were African genotype.

Chikungunya virus (CHIKV) outbreaks have been documented in Africa and Southeast Asia. In India, the first CHIKV outbreak was recorded in 1963 in Calcutta and was followed by epidemics in Chennai, Pondicherry, and Vellore in 1964; Visakhapatnam, Rajmundry, and Kakinada in1965; Nagpur in1965; and Barsi in 1973 (*1*). Recently, CHIKV has emerged in Southeast Asia and the Pacific region (*2**–**4*). Massive outbreaks have been reported from many islands in the Indian Ocean (*5*). *Aedes albopictus* is considered the vector in Reunion and other islands in the Indian Ocean (*5*), but *Ae. aegypti* is the main vector in Asia, including India (*1*). We investigated a large number of patients with fever with arthralgia, reported from October 2005 through March 2006, in many districts from Andhra Pradesh, Karnataka, and Maharashtra states.

## The Study

Blood samples were collected from 1,938 suspected case-patients from the 3 states; serum was separated and transported to the laboratory on wet ice. Adult mosquitoes were collected from houses and sheds. Larval mosquitoes were collected from the affected areas by single-larva survey method. Adult household indexes and Breteau indexes were calculated for each area (*6*).

The C6/36 cell line was used for virus isolation (*7*). Immunoglobulin M (IgM) antibodies to CHIKV (IgM anti-CHIK) and dengue virus (IgM anti-dengue) (*8*) were assayed by IgM capture ELISA. For CHIKV ELISA, brain suspensions from mice infected with CHIKV were the source of antigen, and monoclonal antibodies were the source of antibodies (*9*). Dengue/CHIKV IgM antibodies and negative control human sera were included for respective tests. Approval for use of mice for antigen preparation was obtained from the institutional ethical committee according to national guidelines.

Immunofluorescence assay (IFA) was used to detect the virus in cell culture and in crushed heads of adult mosquitoes (*10*). A patient with the following was confirmed as having CHIKV infection: acute onset of moderate-to-high fever with joint pain of varying severity; negative test results for malaria, typhoid, and tuberculosis; and positive results for IgM anti-CHIKV antibodies, seroconversion, or CHIKV isolation. We used χ^2^ test to compare proportions of cases in different age groups.

We studied CHIKV isolates obtained during current investigations and viruses isolated during earlier epidemics in India (1963–2000) (Table 1). RNA was isolated by using QIAamp Viral RNA Mini Kit (Qiagen, Hilden, Germany) according to the manufacturer's instructions. Superscript II (Invitrogen, Carlsbad, CA, USA) was used for reverse transcription (42°C for 1 h). Initially, *Alphavirus* genus–specific primers that produced a 472-bp fragment (NS4 gene) were F1 5´ GAY GCI TAY YTI GAY ATG GTI GAI GG 3´ and R1 5´ KYT CYT CIG TRT GYT TIG TIC CIGG 3´ (*11*). The second set of primers that amplified a 294-bp product of E1 gene were CHIK/E1-S 5´ TAC CCA TTC ATG TGG GGC 3´ and CHIK/E1-C 5´ GCC TTT GTA CAC CAC GATT 3´ (*12*). For amplification, Platinum *Pfx* enzyme (Invitrogen) was used. Cycling conditions were 1 cycle at 94°C for 5 min; then 35 cycles each of 94°C (1 min), 50°C (1 min), and 68°C (1.5 min); followed by final extension of 7 min at 68°C. The PCR products were purified by using QIAquick PCR Purification Kit (Qiagen) and sequenced by using BigDye Terminator Cycle Sequencing Ready Reaction Kit (Applied Biosystems, Foster City, CA, USA) and an automatic sequencer (ABI PRISM 3100 Genetic Analyzer, Applied Biosystems).

**Table 1:** Chikungunya isolates sequenced, India, October 2005–March 2006

Identification no.	Location, state	Host	Year	GenBank accession no.
IND06KA2	Hajnal village, Karnataka	Human	2006	DQ520739
IND06KA3	Hajnal village, Karnataka	Human	2006	DQ520738
IND06AP4	Kalkada village, Andhra Pradesh	Human	2006	DQ520743
IND06AP6	Kalkada village, Andhra Pradesh	Human	2006	DQ520745
IND06AP5	Mungilipattub, Andhra Pradesh	Human	2006	DQ520744
IND06AP3	Devalammanagaram, Andhra Pradesh	Human	2006	DQ520742
IND06MS2	Kalkada, Andhra Pradesh	Mosquito	2006	DQ520740
IND06MS1	Devalammanagaram, Andhra Pradesh	Mosquito	2006	DQ520741
IND06MH1	Parbhani, Maharashtra	Human	2006	DQ520734
IND06MH2	Parbhani, Maharashtra	Human	2006	DQ520735
IND06MH3	Parbhani, Maharashtra	Human	2006	DQ520736
IND05 KA1	Kotgyal village, Karnataka	Human	2005	DQ520737
IND00MH4	Yawat, Maharashtra	Mosquito	2000	DQ520753
IND73MH5	Barsi, Maharashtra	Human	1973	DQ520752
IND71CH1	Chennai, Tamil Nadu	Human	1971	DQ520751
IND65MH6	Nagpur, Maharashtra	Human	1965	DQ520750
IND65MH7	Nagpur, Maharashtra	Human	1965	DQ520749
IND65AP7	Vishakhapattanam, AP	Human	1965	DQ520754
IND64CH2	Chennai, Tamil Nadu	Human	1964	DQ520748
IND63WB2	Calcutta, West Bengal	Human	1963	DQ520747
IND63WB1	Calcutta, West Bengal	Human	1963	DQ520746

Using ClustalX, version 1.83, multiple alignments of nucleotide sequences were performed. The phylogenic status of the CHIKV isolates was assessed with the software MEGA 3.1 (*13*), Kimura 2-parameter distance, and neighbor-joining algorithm. The reliability of different phylogenic groupings was evaluated with the bootstrap test (1,000 bootstrap replications) available in MEGA.

**Figure 1:**
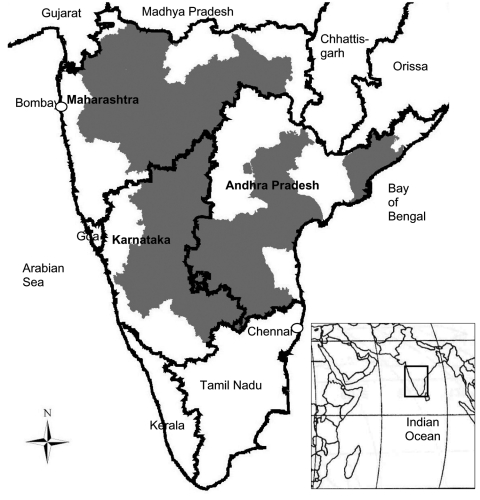
Southern India, 3 states affected by chikungunya virus (October 2005–March 2006). Gray shading indicates area affected in each state.

Acute onset of moderate-to-high fever in association with body ache, backache, and headache was recorded. Joint pain of varying severity occurred within 2 days of onset of fever and, in decreasing order of affliction, involved knees, ankles, wrists, hands, and feet. Joint pain was severe and incapacitating and lasted for weeks to months. Inflammation of joints and transient macular rash on earlobes, neck, trunk, and upper extremities were reported for a few patients. Hemorrhage did not occur. The cases were reported predominantly from rural areas; distribution was focal. Multiple cases were recorded in families. All ages and both sexes were affected; significantly more cases occurred in persons aged >15 years (299 [89.8%] of 333, p<0.001). Cases were reported from 11 of 23 districts in Andhra Pradesh, 15 of 27 in Karnataka, and 16 of 35 in Maharashtra (Figure 1). Results of serologic testing and virus isolation are shown in Table 2.

**Table 2:** Results of serologic testing and virus isolation, India, October 2005–March 2006*

	State		
	Karnataka	Maharashtra	Andhra Pradesh
No. blood samples	900	473	565
Anti-CHIKV IgM, n/N (%)	303/900 (33.7)	169/473 (35.7)	251/565 (44.4)
Anti-dengue IgM, n/N (%)	19/191 (9.9)	23/473 (4.9)	3/325 (0.9)
Anti-CHIKV and anti-dengue IgM, n/N (%)	1/191 (0.5)	2/473 (0.4)	14/325 (4.3)
CHIKV, human serum, n	83	9	20
CHIKV, *Aedes aegypti*, n	4	11	8

*CHIKV, chikungunya virus; IgM, immunoglobulin M.

State governments of Andhra Pradesh, Karnataka, and Maharashtra have declared outbreaks of CHIKV. By mid-April, the declared numbers of fever cases associated with this outbreak were >25,000 in Andhra Pradesh, >65,000 in Maharashtra, and >36,000 in Karnataka. In absence of active surveillance for this disease, these numbers may be underestimates.

The predominant mosquito species in the affected areas was *Ae. aegypti*. *Ae. albopictus* was either absent or present in negligible numbers. The population of *Ae. aegypti* was reasonably high in most of the localities; adult household indexes and Breteau indexes, respectively, were 10–60 and 13–75 in Andhra Pradesh, 20–70 and 40–200 in Karnataka, and 10–30 and 30–50 in Maharashtra. High density of *Ae. aegypti* populations in affected areas and 23 isolations or detections of CHIKV from adult mosquitoes indicate that this species is the main vector in India. Earlier outbreaks in India were mainly restricted to large cities; in contrast, the current outbreak is predominantly rural.

Anti-CHIKV IgM was detected in 33.5% to 41.9% of patients tested. The finding of antibodies to dengue virus in 0.9% to 9.9% of patients and to CHIKV and dengue virus in 0.4% to 4.3% of patients indicates that these viruses cocirculate in the area. Nine patients whose acute-phase serum sample was negative had anti-CHIKV IgM in the early convalescent-phase sample, collected during the second week of illness.

**Figure 2:**
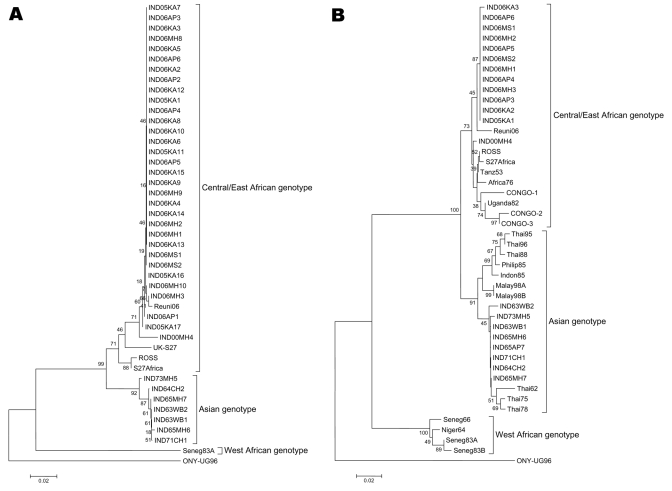
Phylogenic analyses of partial NS4 (456 nt, panel A) and E1 (294 nt, panel B). Refer to Table 1 for the details of the isolates sequenced during this study. Percentage bootstrap support is indicated by the values at each node. The following sequences were obtained from GenBank database: E1, ROSS (AF490259); S27Africa (NC-004162); Tanz53 (AF192905); Africa76 (AF192903); CONGO1 (AY549583); CONGO2 (AY549581); CONGO3 (AY549579); Uganda82 (AF192907); Thai95 (AF192897); Thai96 (AF192900); Thai88 (AF192896); Thai62 (AF192908); Thai75 (AF192898); Thai78 (AF192899); Philip85 (AF192895); Indon85 (AF192894); Malay98A (AF394210); Malay98B (AF394211); Seneg83A (AY726732); ONY-UG96 (AF079456); Reuni06 (DQ443544); Seneg83B (AF192892); Niger64 (AF192893); Seneg66 (AF192891); NS1, UK/S27 (AF345888); ROSS (AF490259); S27Africa (NC-004162); Seneg83A (AY726732); ONY-UG96 (AF079456); and Reuni06 (DQ443544). O'nyong-nyong virus (AF079456) was used as an outgroup.

NS4-based phylogenic analysis identified the Yawat isolate (2000) from Maharashtra as central/East African genotype, not Asian genotype as reported earlier (*14*). This finding led us to resequence all isolates in our repository. Phylogenic analyses based on NS4 (Figure 2A) and E1 regions (Figure 2B) yielded identical results. The Indian viruses isolated from 1963 through 1973 belonged to the Asian genotype, whereas the current isolates from the 3 Indian states and the Yawat isolate belonged to the central/East African genotype. Within the Asian genotype, all older isolates (India 1963–1973 and Thailand 1962–1978) clustered together, whereas later isolates from the Philippines (1985), Indonesia (1985), Thailand (1988, 1995, 1996), and Malaysia (1998) formed a distinct cluster. The sequence from Reunion Islands, which represents a recent outbreak of the disease (GenBank accession no. DQ443544), also grouped with the recent Indian isolates. Percentage nucleotide identity within earlier (1963–1973) and recent (2005–2006) Indian isolates was 99.71% ± 0.16% and 99.94% ± 0.05%, respectively, whereas percentage nucleotide identity between these isolates was 96.11% ± 1.09%. The 2005–2006 Indian isolates were 98.61% ± 0.6% and 98.95% ± 0.57% identical with the Reunion and Yawat isolates, respectively.

## Conclusions

This report confirms CHIKV as the causative agent for large outbreaks of fever with arthralgia and arthritis in 3 Indian states. Thus, chikungunya fever has emerged in outbreak form after 32 years.

The current epidemic is caused by central/East African genotype of CHIKV. That the Yawat isolate is grouped with central/East African genotype suggests that this genotype had been introduced >5 years before the current outbreaks. In this context, determining the genotype of currently circulating strains in Southeast Asia and understanding the modes of transportation of this strain in India and the conditions favoring such large outbreaks would be worthwhile.
